# Dual-band 5G MIMO antenna with enhanced coupling reduction using metamaterials

**DOI:** 10.1038/s41598-023-50446-0

**Published:** 2024-01-02

**Authors:** Daud Khan, Ashfaq Ahmad, Dong-You Choi

**Affiliations:** Information and Communication Engineering, Chosun University, Gwangju, 61452 South Korea

**Keywords:** Aerospace engineering, Electrical and electronic engineering

## Abstract

This article introduces a miniaturized dual-band multiple input multiple output (MIMO) antenna with wide bandwidth and high isolation. The design incorporates ground plane modifications and utilizes metamaterials to achieve dual-band operation in the millimeter wave spectrum for 5G applications, specifically operating at the 28/38 GHz frequency bands. The proposed antenna maintains its dual-band functionality despite its compact size of 3.8 $\times$ 3.7 $\times$ 0.787 $\text {mm}^3$ (without the feed line). The antenna is fabricated on a Rogers RT5880 substrate with a thickness of 0.787 mm and with relative permittivity $\varepsilon _r$ = 2.2. The MIMO system comprises two symmetric radiating elements positioned in close proximity, resulting in mutual coupling levels of $-$ 20 dB and $-$ 12 dB at 25 GHz and 37 GHz, respectively. Modifications are made to the ground length to enhance the isolation at the higher frequency band while embedding metamaterials effectively reduces the coupling at the lower frequency band. The incorporation of metamaterials leads to an enhanced bandwidth from 3.8 to 4.8 GHz in the desired lower band (24–28.8 GHz) and from 3.8 to 4.2 GHz in the higher band (36.6–40.8 GHz). The proposed system can operate across the 28/38 GHz bands using a compact design, thus offering reasonable isolation, an envelope correlation coefficient below 0.0001, and a significant diversity gain (> 9.99 dB). These attributes emphasize the system’s suitability for 5G millimeter-wave cellular communications.

## Introduction

From the past several years, there have been notable developments in wireless communication systems, particularly in regards to their wide bandwidth and high data rates. The development of 5G technology is being propelled by the main factor of supporting a huge number of users with remarkable information rates^1,2^. To address the issue of overcrowded microwave cellular frequencies while implementing 5G systems, the usage of millimeter-wave spectrum has been suggested^3^. The proposal to implement 5G systems using millimeter-wave spectrum aimed at avoiding the problem of congested microwave cellular frequencies. In the 5G spectrum, mobile communications have been assigned a number of frequency bands, including 28, 38, 60, and 73 GHz, which are now in active development and should become commercially viable in the near future^4,5^. It is anticipated that the forthcoming 5G communication systems will meet the ever-increasing requirements of high data rates, reliability, and low power consumption for the billions of connected devices and have the potential to unleash the full capabilities of the emerging technologies like smart cities, virtual reality, and autonomous cars^6^. Researchers face a wide range of challenges when designing antenna’s for millimeter-wave frequencies. In the practical implementation of printed antennas, there are two main design priorities that receive significant attention including bandwidth augmentation and size reduction. Increasing the bandwidth and reducing the physical size are critical objectives when designing printed antenna’s. When it comes to new RF designs, the antenna elements should exhibit specific features such as a compact and low-profile design, support for dual or multiple bands of operation, cost-effectiveness, a modest planar structure, and a minimized physical size^7^.

In addition to the given work, certain other dual-band single-port antennas for millimeter-wave applications are currently undergoing intensive research for the implementation of 5G communication systems. These bands have shown promising advancements in performance and significant improvements compared to previous generations of communication networks^8–14^. Enhancing the quality of transmission in the high-frequency range presents a challenge that can be tackled by integrating alternative approaches, such as multiple-input multiple-output (MIMO) systems. MIMO is commonly acknowledged as a fundamental enabler for 5G communication systems, as it enhances data throughput, maximizes spectral utilization, and optimizes bandwidth efficiency. By leveraging MIMO, the performance and reliability of millimeter-wave transmissions can be significantly improved in the context of 5G networks. This improvement can be achieved by considering a small space between the radiating elements, high isolation, and lower correlation values between the antennas^15–17^. In millimeter-wave systems, the limited space between the antenna’s can result in significant coupling. This coupling has the potential to negatively impact performance of the MIMO system.

Numerous recent studies have been conducted on the development of 5G MIMO antennas operating at millimeter wave (28/38 GHz), which do not use any decoupling techniques^8,18–21^. Consequently, the MIMO system exhibits mutual coupling primarily due to the arrangement of MIMO system. As presented in^8^, there is a coupling at both frequencies of $-$ 22 dB. In contrast^18,19^, demonstrate isolation of 20 dB in the dual-band MIMO system. Additionally, an enhanced isolation of more than 20 dB is presented in^20^. Similarly, the dual-band MIMO system shows improved isolation of 20 dB^21^.

With the rapid advancements in the communication systems, there is an increasing demand for millimeter-wave MIMO antennas with exceptional isolation to cater to the requirements of 5G systems. Additionally, the researcher explored the response of a 5G MIMO single and dual-band antenna, and investigated various decoupling techniques to mitigate the coupling effects^8,16,17^. These antennas present a diverse range of advantages, including miniaturized size, wideband operation, frequency reconfigurability, high gain, circular polarization, and multi-band functionality^22–24^. Researchers have shown significant interest in the application of artificial materials known as metamaterials. These materials have exceptional electromagnetic properties and hold potential for various applications, including antennas and microwave devices, cloaking devices, and super lenses and microscopy^25^. Some reports have explored the use of metamaterials MIMO antennas to achieve mutual coupling reduction and performance enhancement^26–30^. In^26^, the author introduces a metamaterial-based antenna known as DRA (Dielectric Resonator Antenna) with dimensions of 20 $\times$ 40 $\times$ 1.6 $\text {mm}^3$. The antenna achieves an isolation of approximately 29 dB at 28 GHz and possesses diversity parameters of DG (9.96) and ECC (0.05). Another study^27^, utilized a configuration of metamaterial unit cells in a vertical arrangement to reduce coupling at 38 GHz, resulting in improved gains of approximately 6.2 dB and 5.9 dB at 28 and 38 GHz respectively. On the other hand^28^, presents a single-band metamaterial-loaded antenna operating at 6 GHz frequency band, resulting in an improved gain of 8 dB and an isolation of 15.5 dB. To enhance the transmission response, MIMO antennas employ periodic arrangements, making it feasible to achieve maximum transmission efficiency at the resonant frequency band. This can be further improved by using two or more sequentially connected repetitive surfaces^31^. In^29^, a metamaterial-based antenna with dimensions of 28 $\times$ 16 $\times$ 6.3 $\text {mm}^3$ is presented. The antenna operates at 40 GHz and achieves an improved isolation of 33 dB, along with diversity parameters of DG (9.98) and ECC (0.1). Similarly, in^30^, the author introduces a dual-band metamaterial-inspired antenna, resulting in an improved gain of 5.2 dB at 28 GHz and 5.5 dB at 38 GHz, along with enhanced isolation in the MIMO system.

This paper presents an innovative approach to address the challenges of 5G communication by presenting a dual-band MIMO antenna system with wideband capabilities that operates at frequencies of 28 GHz and 38 GHz. From the aforementioned literature, only a few articles discuss the reduction of coupling in dual-band MIMO systems specifically in the millimeter-wave regime. The antenna system comprises of two antennas arranged in close proximity on a Rogers substrate. To enhance the performance of the antenna system, a metamaterial is integrated between the antenna elements. This integration results in a wideband response with improved gain. Furthermore, the integration of metamaterial also helps the proposed design to reduce coupling and enhance isolation between the antennas over the desired frequency band. The exceptional combination of wideband coverage, dual-band operation, enhanced gain, and improved isolation resulting from the integration of metamaterial in this MIMO antenna system highlights its potential to revolutionize high-speed wireless communication in the 5G era. The Ansys High-Frequency Structure Simulator (HFSS) is employed to conduct the simulation.

**Figure 1:**
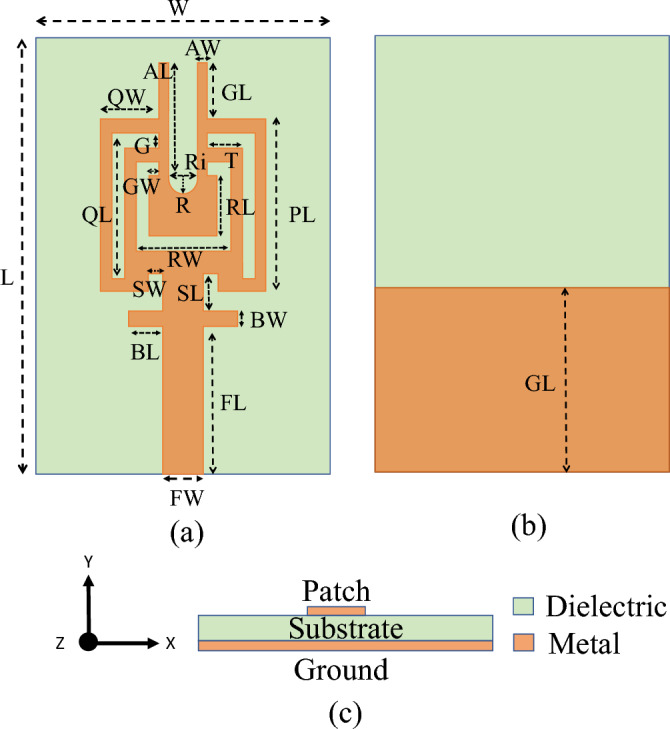
Antenna Geometry in mm: (**a**) Front view. (**b**) Back view (**c**) Side view.

The outline of this paper is structured in the following manner: “Methodology” section presents a comprehensive evaluation of the design methodology for the single-element, MIMO antenna and the metamaterial unit cell. In “MIMO antenna With metamaterial” section delves into the specifics of the MIMO antenna with an integrated unit cell positioned between them, providing various analyses conducted through simulation and measurement. In “MIMO performance parameter” section provides a detailed analysis of the MIMO parameters. In “Comparison” sections present a comparison between the proposed design and recently published state-of-the-art designs. In “Conclusion” section concludes the study, summarizing the findings and discussing the implications of the study.

## Methodology

The main objective of this section is to evaluate the performance of the devised dual-band patch antenna. A comprehensive overview of the stages involved in evaluating the dual-band designs and the unit cell design is provided. Additionally, the findings obtained from electromagnetic simulation and experimental measurements are presented and analyzed. Notably, special emphasis is given to the evaluation of the reflection coefficient for the single element and MIMO antennas without metamaterial. The implications and significance of these results are elucidated through meticulous analysis and in-depth discussion.

**Figure 2:**
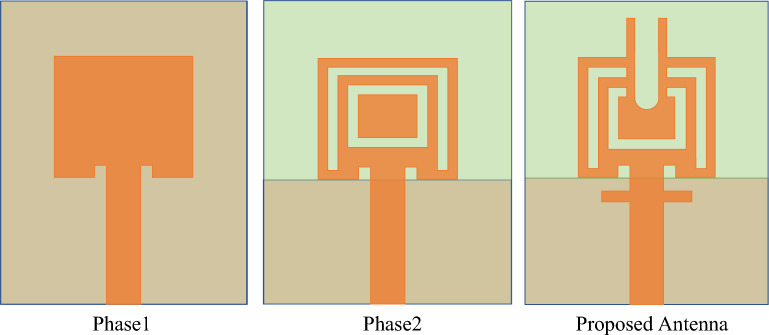
Design evolution of the proposed dual band antenna.

**Figure 3:**
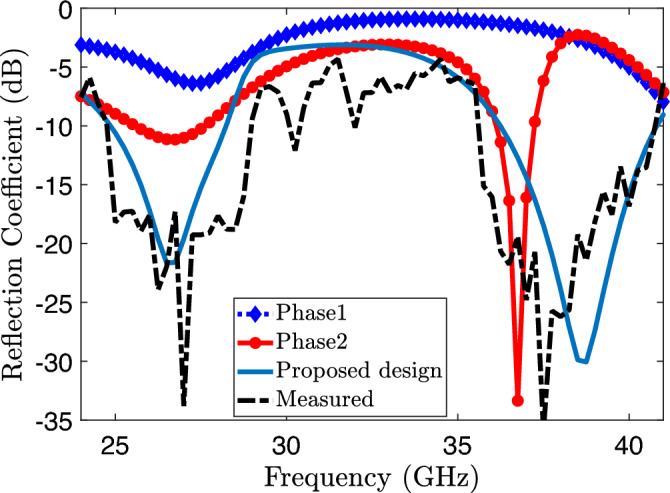
Reflection coefficients ($S_{11}$) for three phases and the measured reflection coefficient of the proposed element.

**Table 1:** Dimension of the proposed single antenna.

Parameter	Dimension (mm)	Parameter	Dimension (mm)
L	9.2	Lc	1.5
W	5	AW	0.2
PL	3.7	Ri	0.6
QW	1.4	R	0.3
QL	3.1	G	0.3
T	0.8	GW	0.2
RL	1.3	RW	2
SW	0.2	SL	0.75
BW	0.3	BL	0.65
FL	3.3	FW	0.9
AL	2.4	GL	1.2

**Figure 4:**
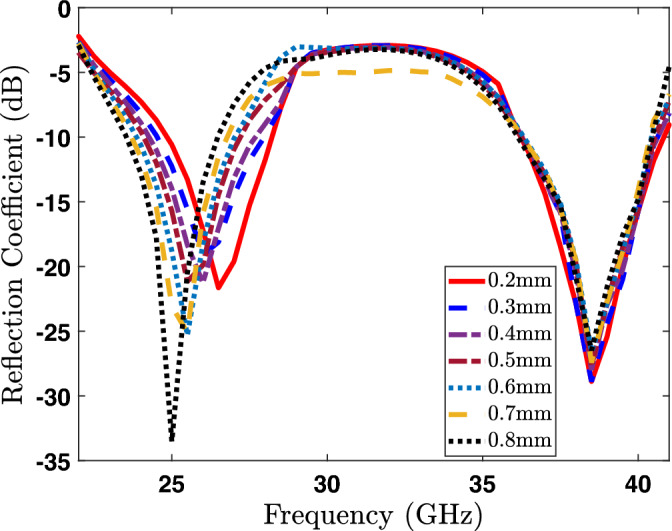
Impact of the slots width on the reflection coefficient ($S_{11}$).

**Figure 5:**
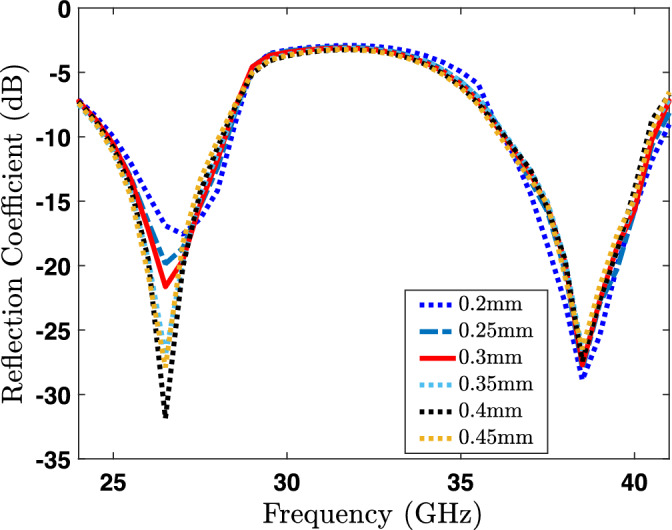
Impact of the branch width on the reflection coefficient ($S_{11}$).

**Figure 6:**
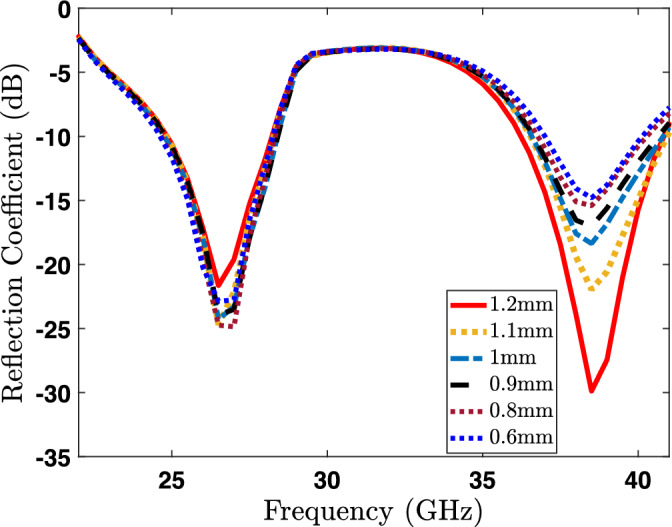
Impact of GL parameter on the reflection coefficient ($S_{11}$).

**Figure 7:**
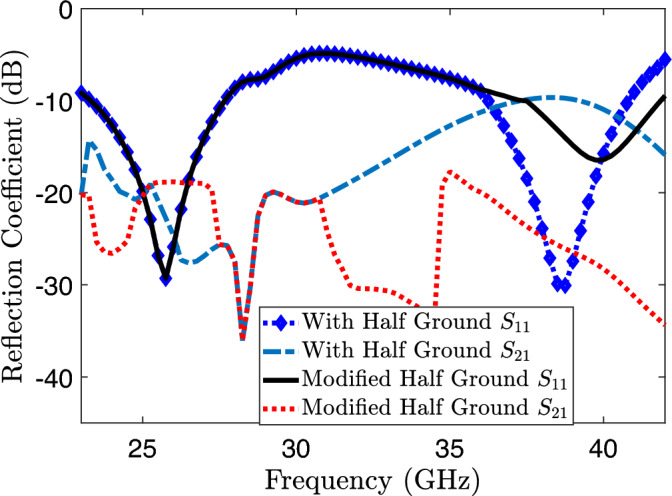
Transmission and reflection coefficients of proposed MIMO system without metamaterial.

### Dual-band design evaluation

A dual-band response is achieved by selecting a low-profile monopole antenna. The antenna is fabricated on an *RT5880* “*Rogers*” substrate with dimensions of 5 $\times$ 9.2 $\times$ 0.787 $\text {mm}^3$. The substrate has a dielectric constant ($\varepsilon _r$) of 2.2 and a loss tangent (tan $\delta$) of 0.0009, as shown in the Fig. 1. The design stages and proposed final design are depicted in the Fig. 2, encompassing the resonator initially inspired by the square patch antenna. The initial phase involved creating a square patch measuring 3.7 mm in length and 3.7 mm in width, thus incorporating a complete ground plane. Additionally, for perfect impedance matching, a pair of slots were inserted near the connection of the patch and the feed line. The reflection coefficient of the particular design is shown in the Fig. 3. To achieve a dual-band and wide-band resonator, the patch must undergo a shape transformation in the next phase. This transformation entails incorporating a couple of slots on the patch and removing half of the ground plane. The resulting resonator exhibits a lower band spanning from 25 to 27.56 GHz and an upper band ranging from 36.3 to 37.8 GHz, as indicated in Fig. 3. Subsequently, the patch is further modified to achieve the desired dual band by adding two identical rods at the top, followed by creating a slot in the same location, resulting in a U-shaped structure.This U-shaped configuration is responsible for the resonance around 28 GHz (24.86–28.65 GHz) and similarly, the branches are connected to the 50 $\Omega$ microstrip line at an angle of 90 degree. The inclusion of identical branches helps achieve the desired upper band, which ranges from 36.24 to 40.82 GHz. Figure 3 shows both the simulated and measured reflection coefficients of the fabricated design. The optimized geometry parameter are mentioned in Table 1.

### Parametric analysis

This section provides a parametric analysis of the proposed antenna. Conducting a parametric analysis on an antenna serves to optimize it and ascertain the most suitable dimensional parameters for the antenna under different scenarios. Again, the proposed dual-band antenna is being set up for examination across a range of parameters to evaluate their influence on the radiator. For our proposed antenna, the crucial parameters to consider in the parametric analysis are the slot width (SW), branches width (BW), and the length of U-shaped rod pairs (GL) at the top of the radiator as shown in the Fig. 1. The parametric analysis concerning slot width (SW) is illustrated in Fig. 4, where the SW parameter is systematically varied from 0.2 to 0.8 mm. The reflection coefficient (S11) demonstrates a noticeable shift of the lower band towards the right side as the width increases from 0.2 to 0.8 mm, as clearly depicted in the Fig. 4. Hence, we can conclude that the resonance frequency of the lower band can be controlled by varying the SW parameter of the radiator. In a similar manner, modifying the BW parameter leads to a variation in the reflection amplitude of the lower band. However, this adjustment has a negligible impact on the resonance frequency of the higher bands, as depicted in Fig. 5. On the other hand, when varying the GL parameter, a noticeable variation in the reflection amplitude of the higher band is observed without affecting the lower band, as depicted in Fig. 6. This observation leads to the conclusion that modifying GL can effectively control the amplitude of the higher band in the proposed antenna.

### MIMO antenna design

A single proposed radiator is strategically placed adjacent to another identical radiator element on the substrate having dimensions 9.2 $\times$ 18 $\times$ 0.787 $\text {mm}^3$ to facilitate the implementation of a MIMO system. This symmetrical arrangement of a two-element MIMO antenna system typically involves placing two antennas in a way that ensures symmetry with respect to axis of the system. The primary principle behind this arrangement is to take advantage of spatial diversity, reduce correlation between antennas, and enhance the reliability and performance of the wireless communication system. Additionally, by reducing the ground length in similar manner to single proposed radiator, the reflection coefficient demonstrates comparable behavior to that of single element. Conversely, the MIMO system experiences mutual coupling at both the lower band (23.8–27.6 GHz) and higher band (36.9–40.4 GHz) as depicted in the Fig. 7. Introducing a square slot at the center of the reduced ground as illustrated in the Fig. 11, responsible for the isolation enhancement in the higher band (36.9–40.4 GHz). However, mutual coupling is still present in the lower band (23.8–27.6 GHz) of the MIMO antenna system, as indicated in Fig. 7. This modification effectively regulates the current distribution over the ground plane, reducing coupling in the higher band of radiating elements. Furthermore, it operates as a band-stop filter in the higher band. After this ground modification, a slight shift towards the right is observed in the higher band, resulting in a frequency range of 37.9 to 41.8 GHz. To improve isolation at higher frequency bands the technique of simple ground modification of a MIMO antenna is used. While the mutual coupling at lower frequency band is eliminated using meta-material.

### Unit cell design

**Figure 8:**
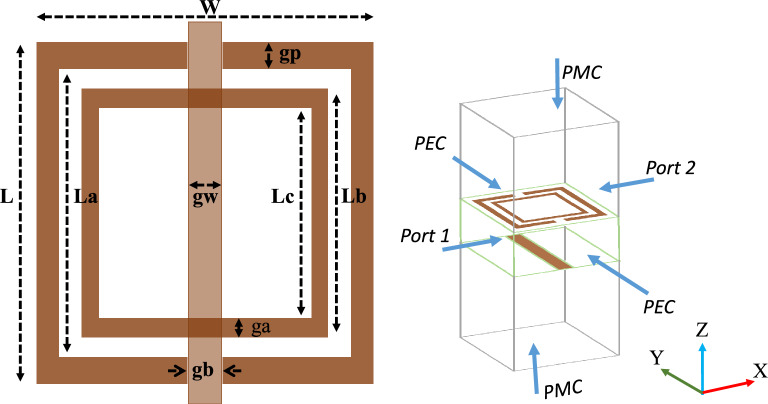
Unit cell analysis (**a**) Unit cell design (**b**) Boundaries arrangement of the unit cell.

The utilization of metamaterials presents a highly effective approach to enhance the radiation performance of antennas, encompassing aspects such as bandwidth, gain and isolation between MIMO radiating elements. The presence of metamaterial significantly effect the surface waves, thus leading to the generation of additional resonance phenomena that contributes to the enhancement of antenna performance^32^. The proposed meta-material unit cell is an altered variant of the split ring resonator (SRR) etched on the Rogers RT5880 substrate with dimensions of 2.7 $\times$ 2.7 $\times$ 0.787 mm$^3$. One side of the substrate consists of square shaped concentric metallic rings, while the other side of the substrate features a dipole wire. The outer metallic ring has couple of splits, and the inner ring does not contain any splits as shown in the Fig. 8a. The geometry parameter of the modified SRR unit cell are specified in the Table 2. To establish a well-defined electromagnetic behavior, the unit cell is surrounded by boundary conditions. In the x-direction, a perfect electric conductor (PEC) boundary is utilized, while a perfect magnetic conductor (PMC) is implemented in the z-direction, creating contrasting boundary arrangements as depicted in the Fig. 8b.

**Figure 9:**
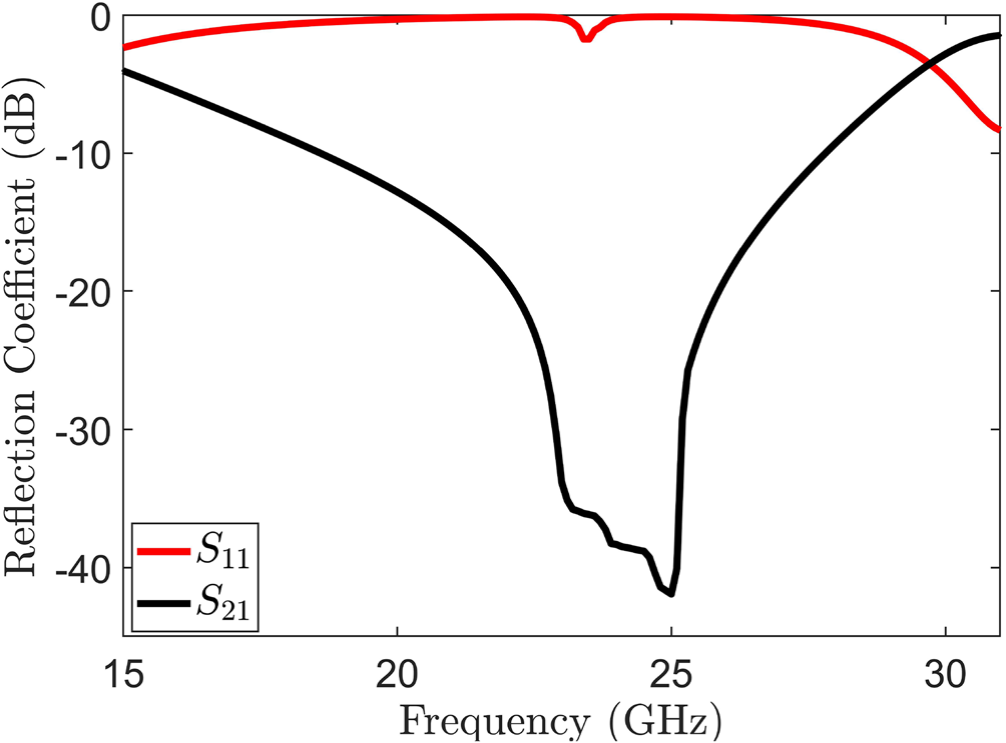
Unit cell transmission and reflection coefficient.

The electromagnetic wave propagates normally in the y-direction, acting as an incident wave that stimulates the resonant structure. Electromagnetic interaction within the unit cell leads to resonance phenomena in the transmitted and reflected waves. The proposed unit cell is designed to operate specifically at 25 GHz to leverage the coupling response at this frequency and improve the isolation of the proposed system. Figure 9 illustrates the transmission and reflection coefficient curves of the unit cell, revealing a pronounced phenomenon of nearly complete reflection at 25 GHz. Furthermore, the proposed unit cell exhibits the ability to function as a band-stop filter within the frequency range of 27 to 29 GHz, which covers the 28 GHz spectrum. The plots vividly demonstrate the capacity of unit cell to attenuate signals within this frequency range, thus effectively serving as a band-stop filter. Furthermore, the reliable retrieval technique is employed to extract the parameters of the metamaterial, including permeability ($\mu$),permittivity ($\epsilon$), and refractive index (n)^33^. This approach utilizes simulated complex S-parameters to calculate the impedance (z) and refractive index (n). The relationship between n, z, and both complex Scattering parameters are defined as follows:

**Table 2:** Dimension of the proposed unit cell.

Parameter	Dimension (mm)	Parameter	Dimension (mm)
L	2.7	Lc	1.5
W	2.7	gb, gp	0.2
La	2.3	gw	0.2
Lb	2	ga	0.25

1 $$\begin{aligned} S_{11} & = \frac{Rz(1 - e^{i2nko d})}{1 - Rz^2 e^{i2nko d}} \end{aligned}$$

2 $$\begin{aligned} S_{21} & = \frac{{(1 - Rz^2) \cdot e^{i2nko d}}}{{1 - Rz^2 \cdot e^{i2nko d}}} \end{aligned}$$

3 $$\begin{aligned} Rz & = \frac{Z-1}{Z+1} \end{aligned}$$

The value of z can be obtained by inverting Eqs. (1) and (2) through the following process:

4 $$\begin{aligned} Z = \sqrt{\frac{{(1 + S_{11})}^2 - (S_{21})^2}{{(1 - S_{11})}^2 - (S_{21})^2}} \end{aligned}$$

where the value of n can be calculated as:

5 $$\begin{aligned} n = 1 - \frac{{kd \left[ \ln (e^{i n k d}) + 2m\pi \right] - i {\text {Re}} \left[ \ln (e^{i n k d}) \right] }}{kd} \end{aligned}$$

Similarly,

6 $$\begin{aligned} e^{i\cdot nko\cdot d} = \frac{S_{21}}{1 - S_{11}Rz} \end{aligned}$$

**Figure 10:**
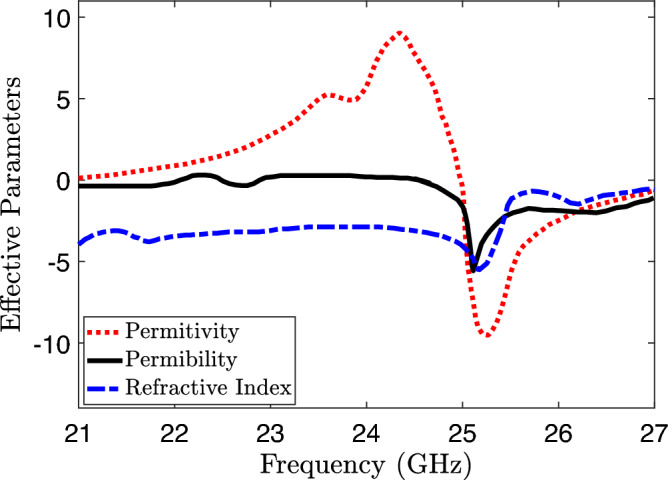
Extracted parameters of the unit cell.

In this context, $k_0$ and *d* are associated with the structure of the metamaterial. One represents the wave number, while the other represents the thickness of the metamaterial. The symbol *m* represents the branch index of *n*. Subsequently, the permittivity ($\epsilon$) and permeability ($\mu$) can be determined by evaluating:

7 $$\begin{aligned} \epsilon & = \frac{n}{z} \end{aligned}$$

8 $$\begin{aligned} \mu & = n \times z \end{aligned}$$

As depicted in the Fig. 10, Eqs. (7)–(8) make it apparent that the permittivity of the unit cell within the desired frequency band is zero, while the permeability also remains zero specifically at 25 GHz. This implies that the material exhibits a refractive index of zero owing to the simultaneous presence of zero permittivity and permeability.

**Figure 11:**
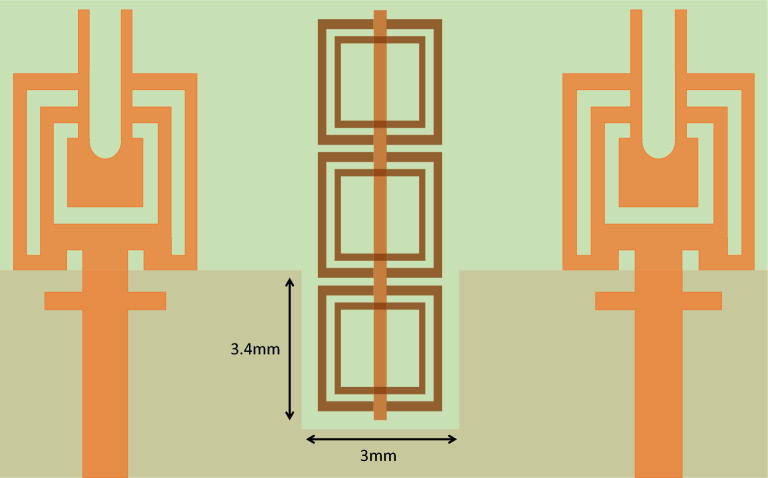
Proposed MIMO design with embedded Metamaterial.

**Figure 12:**
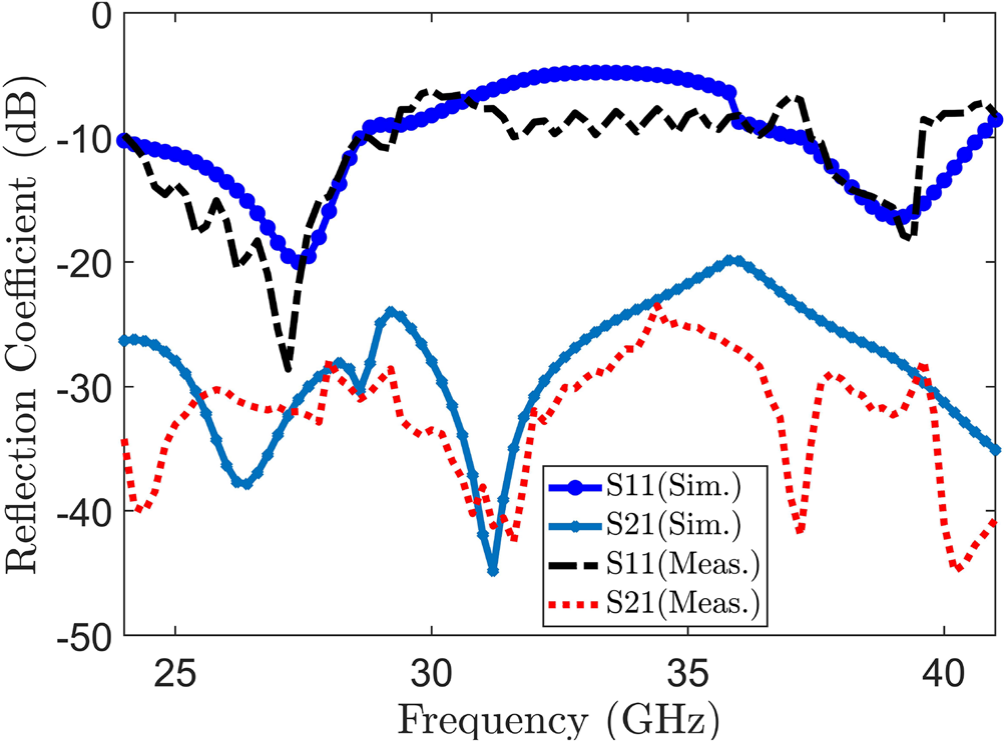
Measured and simulated transmission and reflection coefficient.

**Figure 13:**
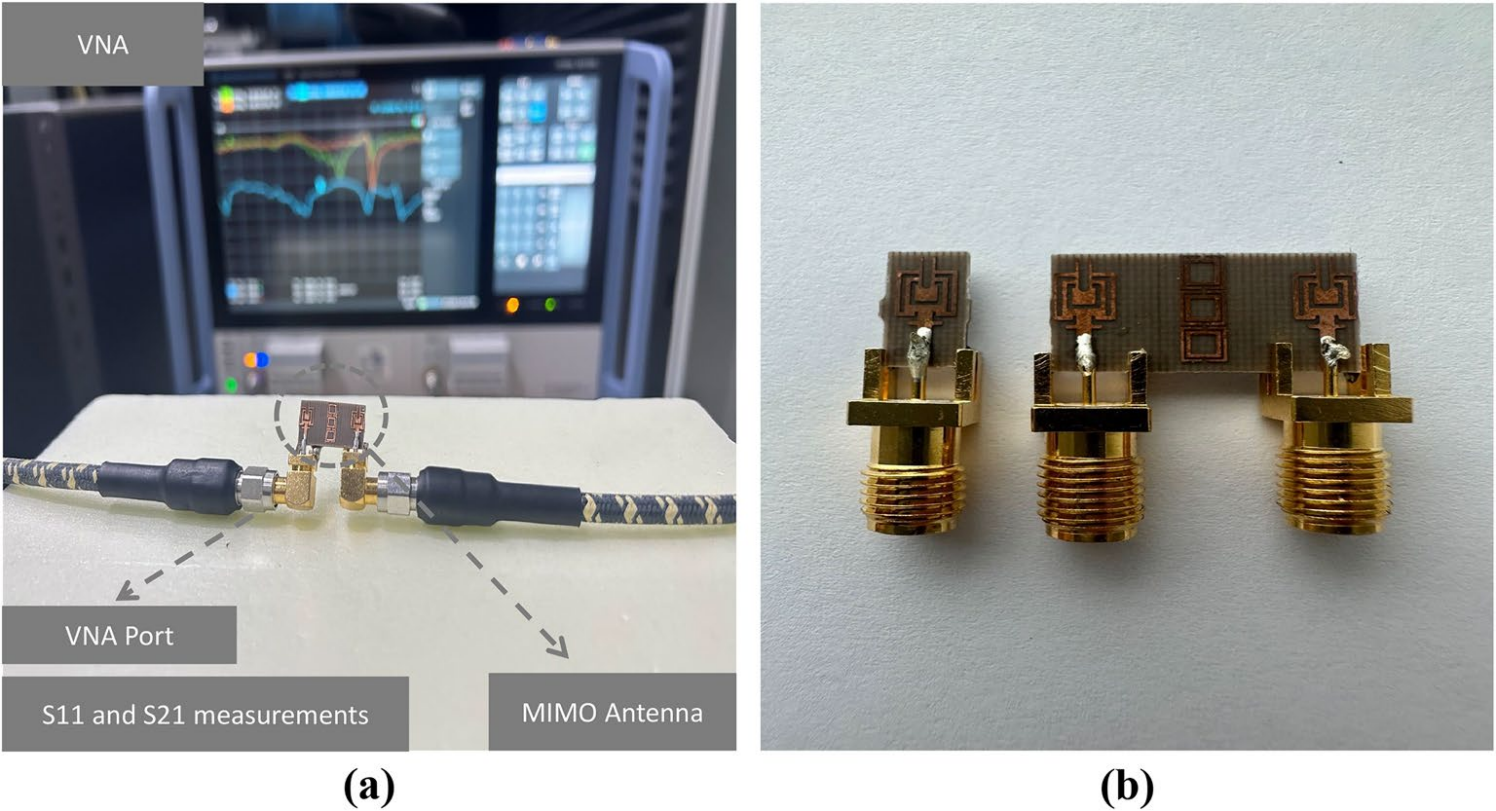
Measuring reflection and transmission coefficients (**a**) Measurement setup (**b**) Fabricated prototype of MIMO design.

**Figure 14:**
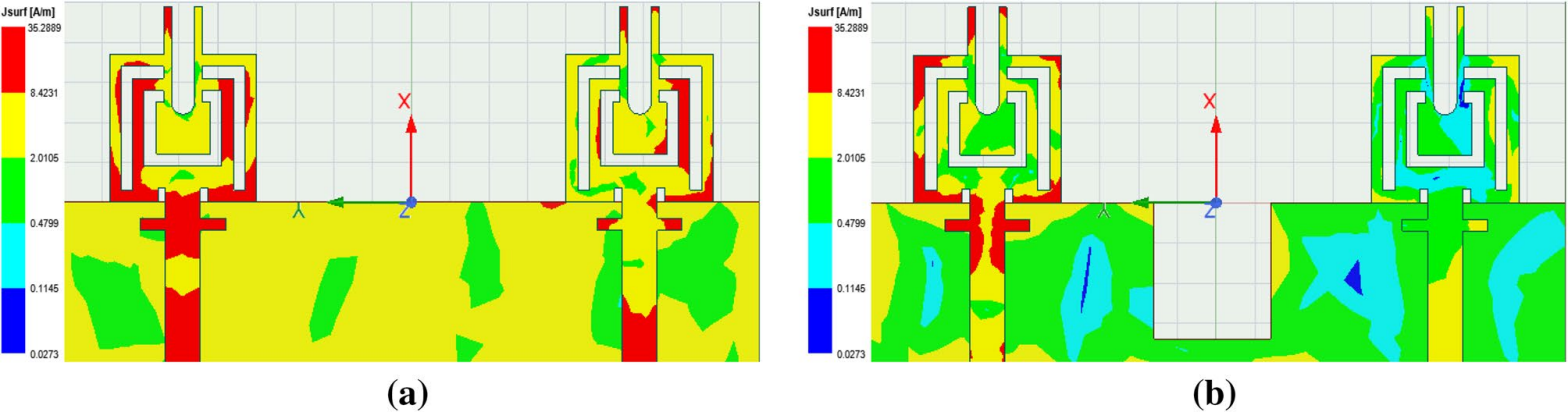
Proposed design surface current distribution at 38 GHz. (**a**) With half-ground (**b**) With modified half-ground.

**Figure 15:**
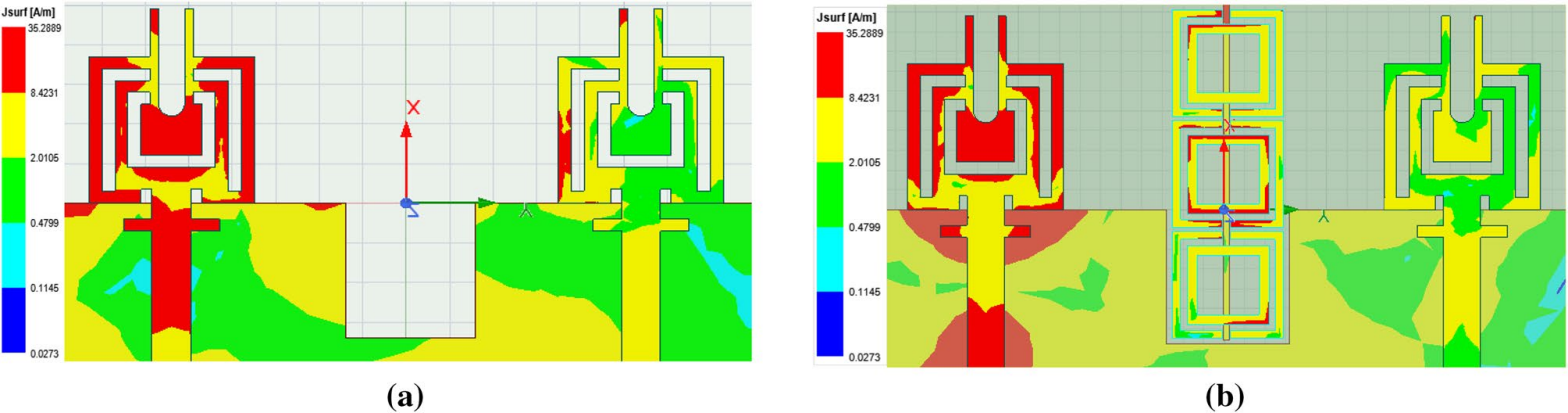
Proposed design surface current distribution at 25 GHz. (**a**) Without metamaterial (**b**) With metamaterial.

## MIMO antenna with metamaterial

The proposed MIMO System experiences mutual coupling at the lower band of 25 GHz. To tackle this challenge, we introduce an array of metamaterial unit cells in a co-planar configuration between the two radiating elements. The geometrical arrangement and excitation technique of this array are meticulously employed to effectively mitigate the coupling effects. As indicated in the Fig. 11, the inclusion of metamaterials in the MIMO antenna configuration results in significant improvements in the bandwidth, and isolation when compared to the setup without metamaterials. Figure 12 presents the reflection and transmission coefficients of MIMO system, considering the modified reduced ground length and inclusion of metamaterial. The incorporation of metamaterials leads to an enhanced bandwidth of 3.8–4.8 GHz in the desired lower band (24–28.8 GHz). Additionally, in the higher band, a slight shift towards lower frequencies is observed in the higher band, resulting in a frequency range of 36.6–40.8 GHz. Furthermore, the comparison of Figs. 7 and 12 a marginal improvement in bandwidth is also observed, spanning from 3.8 to 4.2 GHz. On the contrary, isolation has been improved by incorporating three unit cells on the same substrate between the two elements of the MIMO antenna. This improvement is achieved without increasing the physical separation or making any changes to the antenna orientation and polarization. The metamaterial array is arranged in a way that maximizes its isolation performance in both the frequency bands. This optimization ensures that the desired level of isolation is achieved while working within the constraints of the available physical space, which imposes limitations on the quantity of unit cells that can be utilized. To validate the actual system performance and simulation results, the MIMO antenna incorporating the metamaterial array has been fabricated and tested using a Vector Network Analyzer (VNA), as shown in the Fig. 13. The comparison between the simulated and measured |$S_{11}$| and |$S_{21}$| parameters provides insights into the agreement between the simulated and experimental results. A significant correspondence can be observed between the simulated and measured |$S_{11}$| and |$S_{21}$| datasets, indicating a satisfactory match. However, there are minor variations in the resonant magnitudes between the measured results and the simulation. The surface current distribution of the proposed MIMO antenna is depicted in Figs. 14 and 15, which demonstrates that the antenna with port-1 is powered, while the antenna with port-2 is connected to a 50 $\Omega$ load. Simulation of the MIMO system, initially with an unmodified half ground length, reveals significant mutual coupling at 38 GHz with the neighboring antenna, as shown in Fig. 14a. Conversely, introducing a modification in the form of a square slot at the center of the half ground length mitigating mutual coupling with the neighboring radiator at 38 GHz, as evident in Fig. 14b. Similarly, the surface current distribution is analyzed to provide insight into the role of the metasurface in reducing mutual coupling at 25 GHz. As depicted in the Fig. 15a, in the absence of metamaterial, noticeable mutual coupling is observed at the neighboring antenna, specifically at 25 GHz. On the contrary, when the metamaterial is applied, as shown in Fig. 15b, there is an improvement in the isolation between adjacent antennas. It is observed that the impact of mutual coupling between the adjacent fields is reduced by dispersing the coupled current to the metamaterial layer in a direction opposite to each other within the neighboring rings of the unit cell and adjacent unit cells of the metamaterial. The coupling current distributed to the unit cells of the metamaterial serves as a fundamental technique for enhancing isolation between the MIMO components. As a result, the coupling current between the MIMO components is significantly reduced, leading to a substantial improvement in isolation.

**Figure 16:**
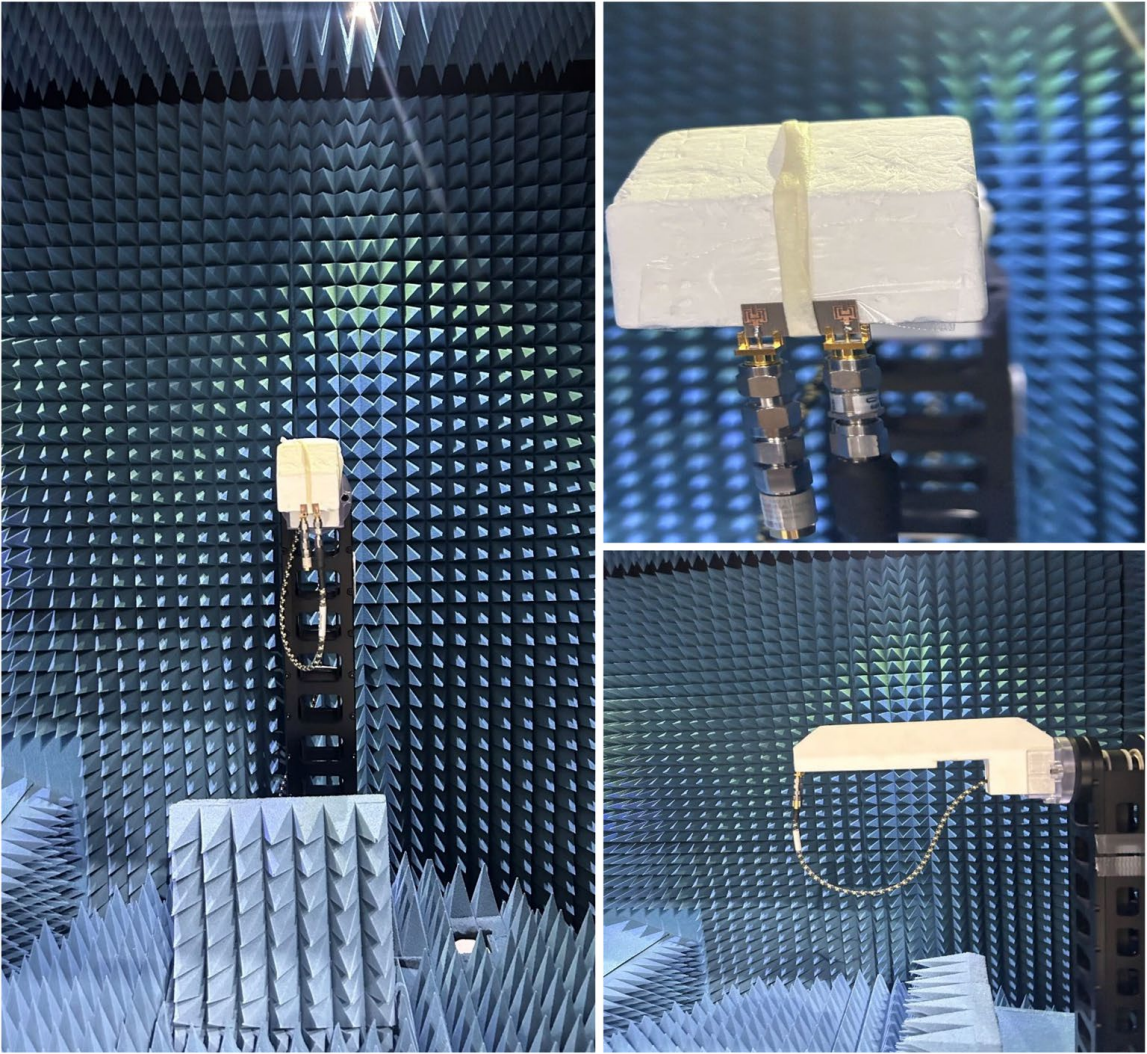
Experimental setup for measuring the radiation pattern and gain of the MIMO.

**Figure 17:**
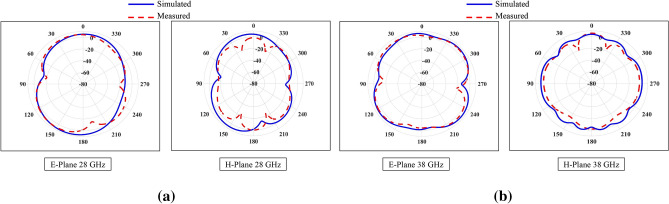
Simulated and measured far-field 2-D gain patterns of MIMO design (**a**) 28 GHz (**b**) 38 GHz.

**Figure 18:**
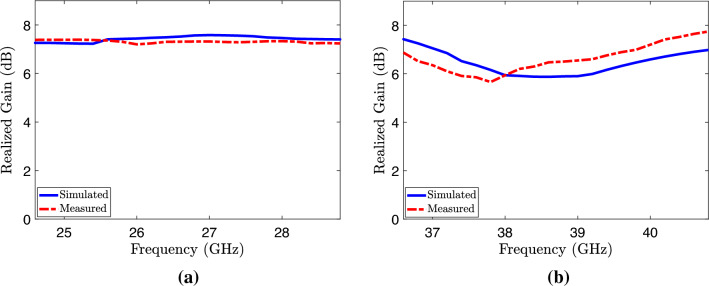
Simulated and measured gain of MIMO design embedded with metamaterial (**a**) Lower bands (**b**) Upper bands.

### Radiation pattern and gain

The radiation patterns are measured and analyzed through a comparative study that involves evaluating the signals received by a standard horn antenna in an anechoic chamber. During the measurement of the radiation pattern, one antenna of the proposed MIMO system is excited, while the other antenna is connected with matched load of 50 $\Omega$ for targeted frequency bands, as depicted in the Fig. 16. In an array environment, this procedure determines how the second element impacts the total gain of the single element. Figure 17 displays the radiation patterns of the MIMO system at 28 and 38 GHz, encompassing both simulated and measured patterns. The measured patterns at port 1 demonstrate that the system is capable of generating a radiation pattern that is predominantly unidirectional. The measured patterns demonstrate a significant degree of concurrence with the simulated patterns in both planes at 28 and 38 GHz. Figure 18 illustrates the simulated and measured plots of the realized gains for the metamaterial-inspired MIMO system. In this configuration, one port is actively energized while the second port is connected to a standard 50-ohm matched load. The lower bands (24–28.8 GHz) demonstrated in Fig. 18a exhibit a simulated realized gain ranging from 7.7 to 7.8 dB. Similarly, the upper band (36.6–40.8 GHz) shown in Fig. 18b displays a simulated realized gain variation between 6 and 7.8 dB. Conversely, the measured gain within the desired frequency bands of 28 GHz and 38 GHz ranges from 7.6 to 7.8 dB. These measured values closely align with the numerical simulation results, albeit with slight deviations. The deviation can be attributed to various factors such as manufacturing tolerances, cable loss, assembly inaccuracies, connector losses, and slight angular deviations in antenna placement within the chamber.

**Figure 19:**
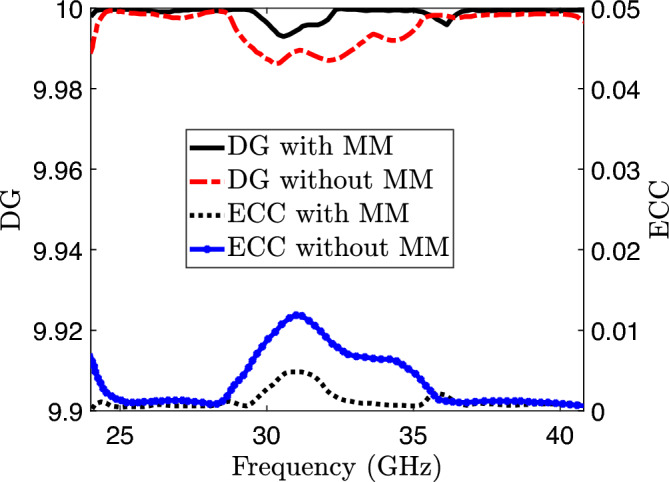
Diversity gain (DG) and envelope correlation coefficient (EEC) with and without metamaterial.

**Figure 20:**
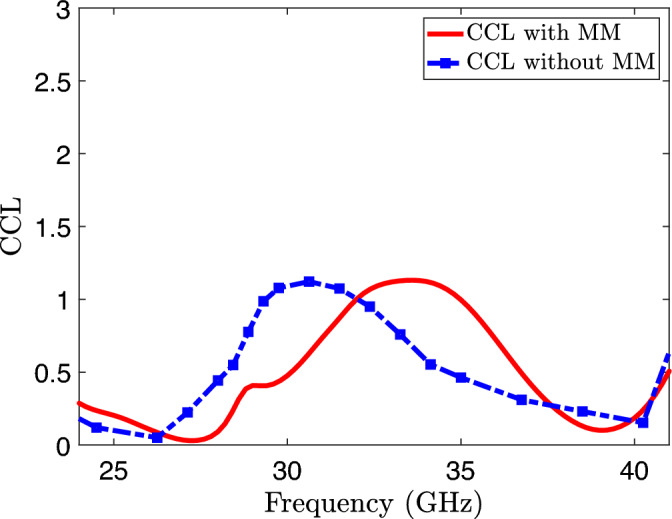
Channel capacity loss of the proposed MIMO system.

## MIMO performance parameter

Assessing the performance of a MIMO system necessitates an evaluation of the correlation characteristics among its ports. Therefore, to evaluate the observed MIMO system and its performance, key parameters including the envelope correlation coefficient (ECC), diversity gain (DG), and channel capacity losses (CCL) are thoroughly investigated. Below, a detailed analysis of these parameters is presented.

### Envelope correlation coefficient and diversity gain

The Envelope Correlation Coefficient (ECC) holds significant importance within MIMO antenna systems. It can be derived from the radiation properties of all antennas in a multi-antenna setup. This coefficient quantifies the impact of a chosen radiating element’s radiation pattern on the radiation characteristics of other elements. In practical applications, the desired value of the Envelope Correlation Coefficient (ECC) is typically $<$ 0.5, while the ideal value is precisely zero. ECC can be determine from the scattering parameter of the two radiating elements as^34^.

9 $$\begin{aligned} \text {ECC} = \frac{{\left| {\textbf{S}}^*_{11} {\textbf{S}}_{12} + {\textbf{S}}^*_{21} {\textbf{S}}_{22} \right| ^2}}{{\left( 1 - \left| {\textbf{S}}_{11} \right| ^2 - \left| {\textbf{S}}_{21} \right| ^2 \right) \left( 1 - \left| {\textbf{S}}_{22} \right| ^2 - \left| {\textbf{S}}_{12} \right| ^2 \right) }} \end{aligned}$$

Figure 19 illustrates the variation of the Envelope Correlation Coefficient (ECC) across the desired frequencies of a proposed antenna system, comparing the cases with and without the inclusion of metamaterials. The observed values reveal that the Envelope Correlation Coefficient (ECC) at 28 and 38 GHz is $<$ 0.0001, demonstrating a significant reduction compared to the practical threshold of 0.5 commonly employed in wireless systems. In MIMO systems, diversity is achieved by receiving multiple transmitted signals through various channel paths, which is made possible by employing multiple antennas in the system. A higher signal-to-noise ratio is attained when the received signals at the transmitter are uncorrelated, resulting in improved signal reception. Mathematically, the diversity gain is determined by referencing to^34^ which indicates that achieving a lower correlation coefficient leads to a higher diversity gain. Figure 19 illustrates that both the frequencies have diversity gain (DG) near the standard value of 10 dB^35^.

10 $$\begin{aligned} \text {DG} = 10 \times \sqrt{1 - |\text {ECC}|^2} \end{aligned}$$

### Channel capacity loss (CCL)

Channel capacity loss is critical factor to consider in multiple-input multiple-output (MIMO) systems. MIMO technology promises higher data rates and improved system performance by utilizing multiple antennas at both the transmitter and receiver. However, in real-world scenarios, channel capacity loss can occur due to various factors. One primary cause is correlated fading, where the wireless channels experience similar fading conditions, reducing the system’s ability to exploit diversity gain. Another factor is spatial correlation, which arises from the proximity of antennas and can lead to a decrease in the number of independent data streams that can be transmitted simultaneously. Additionally, channel capacity loss can occur due to imperfect channel state information at the transmitter, resulting in suboptimal signal precoding and interference management. The channel capacity loss (CCL) of the MIMO system can be computed as:

11 $$\begin{aligned} \text {CCL} = -\log _2(\text {det}(H)) \end{aligned}$$

where H represent correlation matrix,

12 $$\begin{aligned} H & = \begin{bmatrix} \rho _{11} & \rho _{21} \\ \rho _{12} & \rho _{22} \\ \end{bmatrix} \end{aligned}$$

13 $$\begin{aligned} \rho _{ii} & = 1 - \left( |S_{ii}|^2 - |\text {S}_{ij}|^2\right) \end{aligned}$$

14 $$\begin{aligned} \rho _{ij} & = - (S_{ii}^* S_{ij} - S_{ji} S_{jj}^*) \end{aligned}$$

The channel capacity loss (CCL) plots for the MIMO system with and without the inclusion of metamaterial are shown in Fig. 20. At both frequencies, the loaded MIMO system exhibits a CCL of less than 0.05. This value is considerably lower than the accepted threshold of 0.4 bit/s/Hz for wireless systems. The system demonstrates outstanding diversity performance, characterized by remarkably low ECC (less than 0.0001) and CCL (less than 0.05) values, as well as a high DG (approximately 10 dB) at both frequencies. These findings emphasize the suitability of the proposed system for transmission systems that require high data rates.

**Table 3:** Proposed design comparison with recent studies.

References	Size ($\text {mm}^3$)	Frequency of operation (GHz)	Gain (dB)	Isolation (dB)	DG	ECC	CCL (bit/s/Hz)
^26^	20 $\times$ 40 $\times$ 1.6	28 GHz	7.8	29.34	9.96	0.05	Not given
^27^	26 $\times$ 14 $\times$ 0.762	28 and 38 GHz	6.2 and 5.9	34.6 at 28 GHz, 47 at 38 GHz	9.99	0.06	Not given
^28^	80 $\times$ 80 $\times$ 12	6 GHz	8	15.5	9.98	0.004	Not given
^29^	28 $\times$ 16 $\times$ 6.3	40 GHz	10	33	0.98	0.1	Not given
^30^	26 $\times$ 14.5 $\times$ 0.508	28 and 38 GHz	5.2 and 5.5	39 at 28 GHz, 38 at 38 GHz	9.99	0.0001	0.05
^36^	30 $\times$ 15 $\times$ 0.25	28 GHz	5.42	35.8	9.99	0.005	0.1
^37^	20 $\times$ 40 $\times$ 1.6	28 GHz	14.1	35	–	0.01	Not given
^38^	30 $\times$ 35 $\times$ 0.76	28 GHz	8.3	10	9.96	0.05	0.4
^39^	28 $\times$ 28 $\times$ 0.79	28 and 38 GHz	11.65 and 13.65	47.85	9.99	0.001	0.1
^40^	70 $\times$ 40 $\times$ 0.787	4.2 and 4.9 GHz	Not given	25	9.96	0.005	Not given
Proposed work	18 $\times$ 9.2 $\times$ 0.787	28 and 38 GHz	7.8 and 6	30 at 28 GHz, 28 at 38 GHz	9.99	0.0001	0.05

## Comparison

Table 3 provides a comparison between the proposed metamaterial based MIMO antenna and recently developed state-of-the-art MIMO systems. The comparison considered various performance parameters, such as size, bandwidth, gain, isolation, and MIMO performance (ECC, DG, and CLL). The authors of the listed articles in Table 3 presented MIMO systems with different sizes and bandwidths, incorporating enhanced isolation through decoupling structures. In contrast to earlier reported works, the suggested MIMO system with a metamaterial outperforms them in the field of bandwidth, gain and isolation. Furthermore, the developed MIMO system showcases exceptional diversity performance and overall efficiency while maintaining compact dimensions compared to related reported antennas. While the antennas reported in^30^ do offer little higher isolation compared to our suggested MIMO design, they suffer from large size, low gain, and narrow bandwidth compared to the our proposed MIMO system. Furthermore, the proposed miniaturized system demonstrates better isolation, gain and bandwidth. In the 5G era, the presented dual-band MIMO system based on metamaterials is exceedingly well-suited for the millimeter-wave communication system.

## Conclusion

A miniaturized MIMO antenna design featuring a metamaterial array is presented. This design allows for operation in dual millimeter-wave bands while effectively managing low mutual coupling. The incorporation of this antenna configuration specifically caters to the needs of advanced 5G communication networks. The proposed MIMO design achieves a dual-band response at 28/38 GHz and consists of two adjacent radiating elements with a compact physical size of 9.2 $\times$ 18 $\times$ 0.787 $\text {mm}^3$. To enhance isolation without altering the system’s footprint, a well-designed dual-band metamaterial is strategically placed in the midst of the two radiating elements of the MIMO System. Exceptional performance in the millimeter-wave spectrum is assured by implementing both the MIMO antenna system and metamaterial on the Rogers RT5880 low-loss substrate. Furthermore, the MIMO system exhibits remarkable diversity characteristics, featuring an exceptionally low ECC (< 0.0001) and CLL (< 0.05 bit/s/Hz), DG (> 9.99 dB), and an omnidirectional radiation pattern. The simulated results closely correspond to the measured outcomes across all system performance metrics. The proposed antenna system possesses these advantages, firmly positioning it as a viable choice for 5G millimeter-wave communication systems.

## Data Availability

All the data generated or analyzed during this study are included in this study are included in this published article.
